# Caffeic Acid Phenethyl Ester and Caffeamide Derivatives Suppress Oral Squamous Cell Carcinoma Cells

**DOI:** 10.3390/ijms24129819

**Published:** 2023-06-06

**Authors:** Yin-Hwa Shih, Chieh-Chieh Chen, Yueh-Hsiung Kuo, Lih-Jyh Fuh, Wan-Chen Lan, Tong-Hong Wang, Kuo-Chou Chiu, Thanh-Hien Vu Nguyen, Shih-Min Hsia, Tzong-Ming Shieh

**Affiliations:** 1 Department of Healthcare Administration, Asia University, Taichung 41354, Taiwan; 2 School of Dentistry, China Medical University, Taichung 40402, Taiwanngvuthanhhien@gmail.com (T.-H.V.N.); 3 Department of Chinese Pharmaceutical Sciences and Chinese Medicine Resources, China Medical University, Taichung 40402, Taiwan; 4 Department of Biotechnology, Asia University, Taichung 41354, Taiwan; 5 Chinese Medicine Research Center, College of Chinese Medicine, China Medical University, Taichung 40402, Taiwan; 6 Department of Dentistry, China Medical University Hospital, Taichung City 404332, Taiwan; 7 Tissue Bank, Chang Gung Memorial Hospital, Taoyuan 33305, Taiwan; 8 Division of Oral Diagnosis and Family Dentistry, Tri-Service General Hospital, National Defense Medical Center, Taipei 11490, Taiwan; 9 School of Nutrition and Health Sciences, Taipei Medical University, Taipei 110301, Taiwan; 10 Nutrition Research Center, Taipei Medical University Hospital, Taipei 110301, Taiwan; 11 Department of Dental Hygiene, China Medical University, Taichung 40402, Taiwan

**Keywords:** autophagy, caffeic acid phenethyl ester (CAPE), caffeamide derivative, oral squamous cell carcinoma (OSCC), reactive oxygen species (ROS)

## Abstract

Caffeic acid phenethyl ester (CAPE) contains antibiotic and anticancer activities. Therefore, we aimed to investigate the anticancer properties and mechanisms of CAPE and caffeamide derivatives in the oral squamous cell carcinoma cell (OSCC) lines SAS and OECM-1. The anti-OSCC effects of CAPE and the caffeamide derivatives (**26G**, **36C**, **36H**, **36K**, and **36M**) were evaluated using the 3-(4,5-dimethylthiazol-2-yl)-2,5-diphenyltetrazolium bromide test. Cell cycle and total reactive oxygen species (ROS) production were analyzed using flow cytometry. The relative protein expression of malignant phenotypes was determined via Western blot analysis. The results showed that **26G** and **36M** were more cytotoxic than the other compounds in SAS cells. After **26G** or **36M** treatment for 48 h, cell cycle S phase or G2/M phase arrest was induced, and cellular ROS increased at 24 h, and then decreased at 48 h in both cell lines. The expression levels of cell cycle regulatory and anti-ROS proteins were downregulated. In addition, **26G** or **36M** treatment inhibited malignant phenotypes through mTOR-ULK1-P62-LC3 autophagic signaling activated by ROS generation. These results showed that **26G** and **36M** induce cancer cell death by activating autophagy signaling, which is correlated with altered cellular oxidative stress.

## 1. Introduction

Oral cancer is a common type of head and neck cancer, with an estimated 650,000 new cases reported annually worldwide. Oral squamous cell carcinoma (OSCC) is a malignant tumor originating from squamous cells lining the oral cavity, such as the lips, hard palate, gums, tongue, and cheeks. The prevalence of OSCC varies with geographic location, with higher rates observed in some areas of Asia, South America, and Africa. A previous study indicated that oral cancer is highly prevalent in Asia-Pacific men, owing to the use of tobacco, alcohol, and areca nuts (betel quid) in these regions [1]. Unlike in North America and Europe, human papillomavirus (HPV) infection is a major risk factor in oropharyngeal cancer (OPC) [2]. In clinical practice, standard treatments for oral cancer include surgery, radiation therapy, chemotherapy, targeted therapy, immunotherapy, and reconstructive surgery. The best treatment plan varies depending on individual case conditions, such as the stage and location of cancer and the patient’s overall health and preferences. Generally, the 5-year survival rates of patients with early-stage (I and II) and advanced-stage (III and IV) OSCC are about 83–90% and 50–60%, respectively. However, some studies report that the highest survival rate reached only 56–68% on average in patients with oral cancer within 5 years [3,4]. Therefore, there is an urgent need to develop more effective methods for treating oral cancer.

Propolis is a natural substance produced by bees and is commonly used in traditional medicine because of its health benefits, such as antimicrobial activity, anti-inflammatory effects, immune system support, and antioxidant properties [5]. Propolis products exist in various forms, such as tinctures, ointments, creams, sprays, and capsules. In addition, propolis can be used to support oral health by promoting gum health and reducing plaques and bad breath. The bioactive ingredients of propolis include flavonoids, phenolic acids, esters, terpenoids, steroids, and various amino acids [6]. Caffeic acid phenethyl ester (CAPE) is the major bioactive compound in propolis [7] and may be effective in treating various types of cancers, including breast, colon, lung, and prostate cancers [8].

The exact mechanism by which CAPE exerts its antitumor effects is not fully understood but is believed to involve the regulation of multiple signaling pathways related to cancer cell growth and survival. Previous studies have indicated that the anticancer effects of CAPE on oral cancer involve mediation of apoptotic signaling [9] or increased N-myc downstream-regulated gene 1 (NDRG1) expression to suppress cancer cell growth via mitogen-activated protein kinase (MAPK) signaling [10]. Moreover, CAPE exerts its antimetastatic effects in oral cancer by downregulating the expression of matrix metalloproteinase-2 (MMP-2) following the failure of focal adhesion kinase (FAK) and MAPK signaling activation [11]. These scientific reports showed that CAPE could be used as a clinical drug for treating patients with oral cancer.

Hence, we speculated that CAPE could potentially treat oral cancers. However, CAPE is poorly soluble in aqueous environments and is hydrolyzed to caffeic acid by esterases in vivo [12,13], which limits CAPE applications in cancer therapy. CAPE has been found to possess antimitogenic, anticarcinogenic, anti-inflammatory, and immunomodulatory properties in in vitro studies [14]. Additionally, it has demonstrated antibacterial activity. Previous research has suggested the potential of CAPE as a therapeutic agent for various cancers, including prostate cancer [15], osteosarcoma [16], breast cancer [17] and others. Considering this, we believe that CAPE could be a potential candidate for the treatment of oral cancer. To the best of our knowledge, there are limited studies investigating the effects of CAPE and its derivatives on anti-OSCC (oral squamous cell carcinoma). Hence, we are particularly interested in exploring the potential of these compounds in combating oral diseases. To explore a more stable CAPE, caffeamide derivatives were designed, synthesized, and pharmacologically assessed in vitro to evaluate their anti-oral cancer potential. This study aimed to identify the potential anti-oral cancer compounds from CAPE and four caffeamide derivatives and analyze the mechanisms triggering cell death in OSCC cells.

## 2. Results

### 2.1. The Cytotoxicity of ***26G*** and ***36M*** in Oral Cancer Cell Lines

First, the cytotoxicity of the CAPE and 5 caffeamide derivatives (**26G**, **36C**, **36H**, **36K**, and **36M**) was tested in the SAS cell line. The cells were treated with the vehicle or 12.5–200 μM of each compound for 24 h to determine the half-maximal inhibitory concentration (IC50). The chemical structures, molecular weights, and IC50 values of the SAS cell lines are presented in Table 1. The IC50 values of **36C**, **36K**, and **36H** were greater than 200 μM. SAS cells were more sensitive to **26G** and **36M** than to the other compounds. Therefore, this study focused on analyzing the anticancer potentials of **26G** and **36M**.

**Figure 1:**
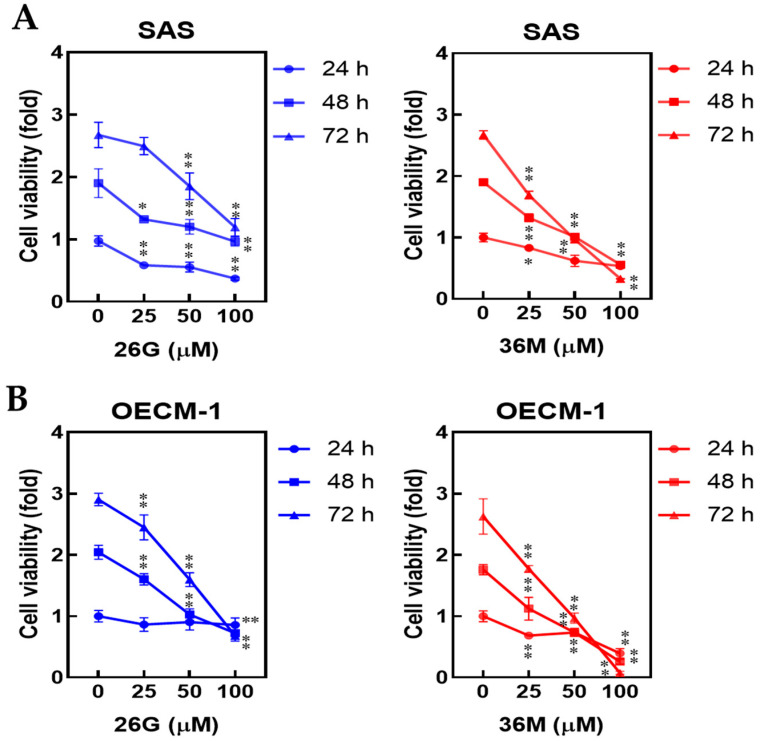
The **26G** and **36M** decreased cell viability in dose- and time-dependent manners in oral cancer cells. (**A**) SAS and (**B**) OECM-1 cells were treated with **26G** or **36M** doses at 25, 50, and 100 μM for 24, 48, and 72 h. The cell viability was determined using the MTT method. * *p* < 0.05, ** *p* < 0.01 compared with the vehicle control group. MTT, 3-(4,5-dimethylthiazol-2-yl)-2,5-diphenyltetrazolium bromide.

The dose- and time-dependent cytotoxicities of **26G** and **36M** were analyzed in SAS and OECM-1 cell lines. Both SAS and OECM-1 cells showed a decreased cell viability rate upon treatment with **26G** and **36M** at 25, 50, or 100 μM for 24, 48, and 72 h, compared to that of the vehicle control group (Figure 1). The cytotoxicity of **26G** and **36M** were dose- and time-dependent increased in the SAS cell line (Figure 1A) and OECM-1 (Figure 1B). The inhibitory effect on cancer cells showed that **36M** was better than **26G** for 48 and 72 h, although the IC50 of **26G** was lower than the IC50 of **36M** (Table 1). Based on the cell viability rate at the same dose, we found that **36M** exhibited more powerful anticancer ability than **26G** did by inducing cell toxicity. SAS cells were more sensitive to **26G** treatment for 24 h than OECM-1 cells were. However, the cytotoxicity of **26G** was stronger for OECM-1 at 48 and 72 h.

### 2.2. The ***26G*** and ***36M*** Treatments Increased G1 Phase and S Phase Arrest

**Figure 2:**
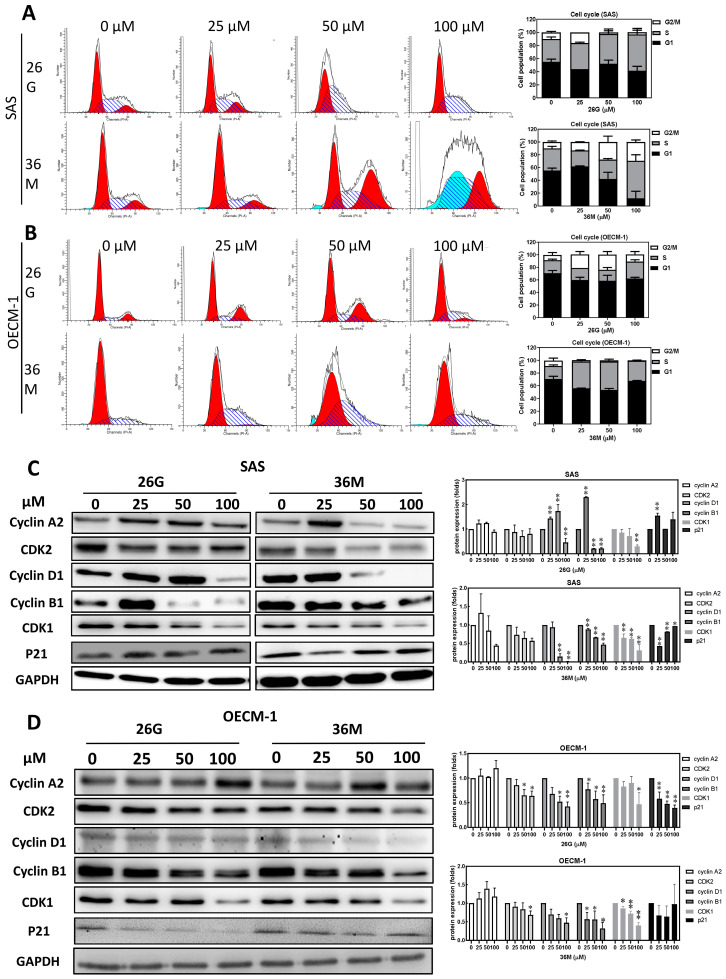
**26G** and **36M** treatments increased S and G2/M phase arrest in OSCC cell lines. OECM-1 cells were treated with 25–100 μM **26G** or **36M** for 48 h. (**A**,**C**), SAS; (**B**,**D**), OECM-1. (**A**,**B**), histograms plotted by analyzing changes in cell cycle progression by performing flow cytometry analysis. The vertical axis represents the cell number, and the horizontal axis represents the PI staining strength. Cell cycle distribution is shown in the bar graph. The vertical axis represents the percentage of cells in the G2/M, S, and G1 phases of the cell cycle, and the horizontal axis represents the concentration of **26G** and **36M** used. (**C**,**D**), the CDK1, cyclin A2, CDK2, cyclin D1, cyclin B1, and P21 expression determined by Western blotting analysis. GAPDH was used as the internal control. The cell cycle-related proteins were quantified, and the results are presented in the right histogram. Data are expressed as mean ± SD; * *p* < 0.05, and ** *p* < 0.01 compared to the vehicle control group. SD, standard deviation.

Analyzing the effects of **26G** and **36M** on the cell cycle is important for understanding their anticancer mechanisms. PI staining and flow cytometry were performed to analyze whether **26G** or **36M** treatment induced cell cycle arrest. The cell cycle was arrested in the SAS and OECM-1 cells at the S or G2/M phase after treatment with **26G** and **36M** for 48 h. The **26G** induced S phase arrest and **36M** induced S and G2/M phase arrest in SAS cells (Figure 2A). The S and G2/M phase arrest was induced by **26G**, and **36M** induced S phase arrest in OECM-1 cells (Figure 2B). Western blotting was performed to analyze the relative protein expression during the cell cycle. The results showed that both SAS and OECM-1 cells treated with **26G** or **36M** at 100 μM significantly decreased the protein expression of CDK1, CDK2, cyclin D1, and cyclin B1 compared with the control group. Cyclin A2 expression was inhibited by **26G** and **36M** in SAS cells but not in OECM-1 cells. P21 was upregulated in SAS cells but downregulated in OECM-1 cells (Figure 2C,D). The occurrence of cell cycle-related protein downregulation supports the result of 100 μM **26G** and **36M** inducing S and G2/M phase arrest in SAS and OECM-1 cells. However, 25 μM **26G** treatment enhanced cyclin D1 and cyclin B1 expression (Figure 2C) and the cell viability was not enhanced (Figure 1A), which may be related to the antioxidant capacity of the cells [18].

### 2.3. After ***26G*** and ***36M*** Treatment, ROS Increased at 24 h and then Decreased at 48 h

**Figure 3:**
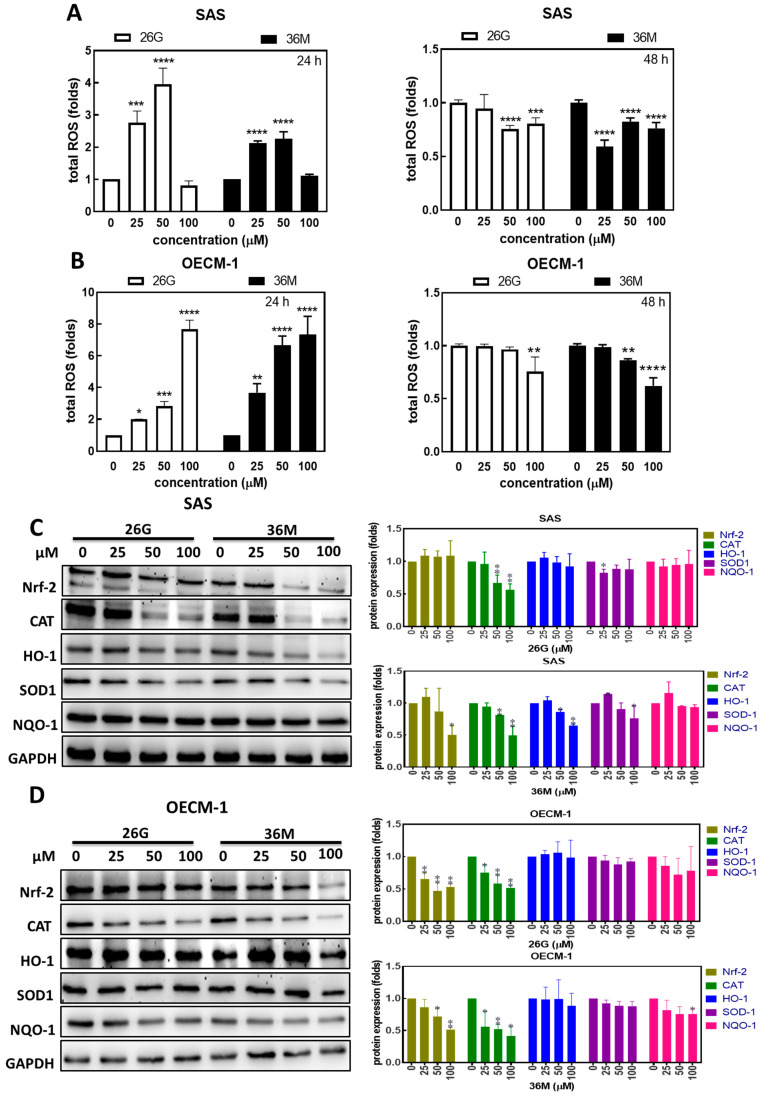
Treatment with **26G** and **36M** increased ROS production after 24 h treatment, then decreased ROS after 48 h treatment in OSCC cell lines. The OSCC cells were treated with 25–100 μM **26G** or **36M** for 24 h and 48 h. The fold changes in ROS production in SAS (**A**) and OECM-1 (**B**). Left panels, 24 h; right panels, 48 h. After **26G** or **36M** treatment for 48 h, the protein expressions of Nrf-2, CAT, HO-1, SOD-1, and NQO-1 in SAS (**C**) and OECM-1 (**D**) were analyzedvia Western blotting. The protein expression level quantification of each targeted protein under each treatment is shown in the histograms on the right. GAPDH was used as a loading control. * *p* < 0.05, ** *p* < 0.01, *** *p* < 0.001, **** *p* < 0.0001 compared to the vehicle control group.

CAPE is an antioxidant. However, whether or not **26G** and **36M** have antioxidant activities remains unclear. Total ROS stress was measured using the carboxy-H2DCFDA detection method. The ROS levels increased under the 25 μM and 50 μM **26G** or **36M** treatment, and decreased under the 100 μM **26G** or **36M** treatment for 24 h in SAS cells. However, the ROS levels decreased under 25 μM to 100 μM **26G** or **36M** treatment for 48 h in SAS cells (Figure 3A). In OECM-1 cells, the ROS levels increased under the 25–100 μM **26G** or **36M** treatment for 24 h but decreased under 100 μM **26G** or 50 μM, and 100 μM **36M** treatment for 48 h (Figure 3B). Overall, treatment with **26G** below 50 μM or **36M** below 100 μM for 24 h induced ROS generation in SAS and OECM-1 cells, respectively. SAS and OECM-1 cells showed similar trends in ROS production after **26G** and **36M** treatment, but SAS was more sensitive than OECM-1 was. The antioxidant activity of **36M** was better than that of CAPE (**26G**) in both SAS and OECM-1 cells. Next, we analyzed the expression levels of antioxidant-associated proteins in both cell lines after treatment with **26G** and **36M** for 48 h. Nrf-2, CAT, HO-1, and superoxide dismutase type 1 (SOD-1) levels showed a decreasing trend in SAS cells treated with **26G** and **36M** (Figure 3C, left panel). Treatment with **26G** significantly reduced CAT expression levels and treatment with **36M** reduced Nrf-2, CAT, and HO-1 expression levels in SAS cells (Figure 3C, histograms). The Nrf-2, CAT, and NQO-1 also show a weakening trend in OECM-1 cells treated with **26G** and **36M** (Figure 3D, left panel). The **26G** treatment significantly reduced Nrf-2 and CAT expression, and the **36M** treatment reduced Nrf-2, CAT, and NQO-1 in OECM-1 cells (Figure 3D, histograms). Although antioxidant proteins were inhibited by the **26G** and **36M** treatments for 48 h in SAS and OECM-1 cells, total ROS increased at 24 h and then decreased at 48 h. These data show that OSCC cells were not protected by **26G** and **36M** antioxidant mechanisms. It has been speculated that **26G** and **36M** neutralize free radicals by accepting or donating electrons (s) to eliminate the unpaired condition of the radical [19]. A previous study suggested that patients with low ROS levels may be more sensitive to anticancer therapy [20]. Therefore, **26G** or **36M** may improve the efficacy of chemotherapy, but this needs to be confirmed in in vivo models.

### 2.4. Treatment with ***26G*** and ***36M*** Induced Autophagy Via mTOR-ULK1-P62-LC3 Signaling Caused by ROS Generation

**Figure 4:**
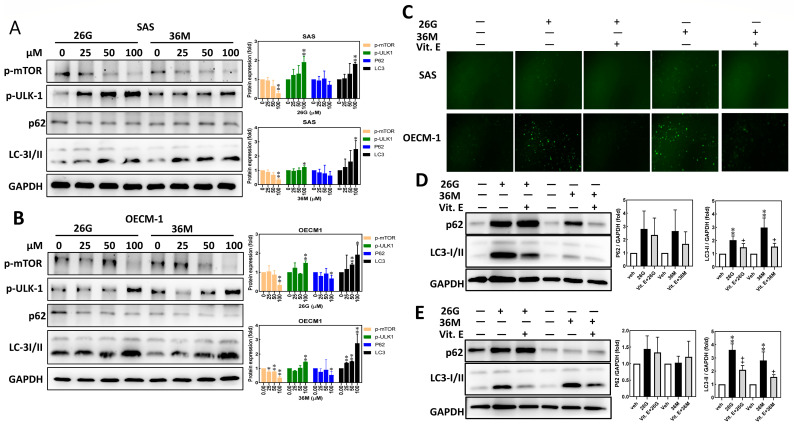
The **26G** and **36M** treatment for 24 h activated mTOR-ULK1-P62-LC signaling by ROS production in OSCC cell lines. The OSCC cells were treated with **26G** or **36M** for 24 h. Subsequently, the autophagy-relative protein expressions of p-mTOR, p-ULK-1, p62, and LC-3 I/II in SAS (**A**) and OECM-1 (**B**) were determined by western blot. The protein expression level quantification under each treatment is shown in the right histograms. The SAS or OECM-1 cells were pretreatment with vitamin E (20 μM) for 2 h, and the **26G** or **36M** (100 μM) was then added for another 24 h (**C**–**E**). (**C**) The intracellular ROS level was analyzed by the DCFDA approach. Fluorescence images were recorded under 100X magnification using a fluorescence microscope. Up panel, SAS; low panel, OECM-1. The protein expressions of LC3 in SAS (**D**) and OECM-1 (**E**) were determined by western blot, and the protein expression level quantification under each treatment is shown in the right histograms. The GAPDH was used as a loading control. * *p* < 0.05, ** *p* < 0.01 compared to the vehicle control group. + *p* < 0.05, ++ *p* < 0.01 compared to the **26G** or **36M** group alone.

ROS control and determine the type of cell death including autophagy, apoptosis, ferroptosis, and necroptosis [21]. CAPE induces autophagy in human SH-SY5Y neuroblastoma cells [22], breast cancer MDA-MB-231 cells [23], and colorectal adenocarcinoma WiDr cells [24]. In the OSCC cell lines, SAS and OECM-1, treatment with **26G** and **36M** increased the levels of phosphorylated ULK-1 and LC3-II proteins in a dose-dependent manner. However, the phosphorylated mTOR and p62 remarkably decreased after the 25–100 μM of **26G** and **36M** treatment in SAS (Figure 4A) and OECM-1 (Figure 4B) cells. Vitamin E (VE) is a common antioxidant. Pretreatment with vitamin E reduced the **26G** and **36M**-stimulated total ROS signals (Figure 4C). Briefly, **26G** and **36M** down-regulated protein expressions; LC-3 and P62 were reversed by vitamin E pretreatment in SAS (Figure 4D) and OECM-1 cells (Figure 4E). These results suggest that **26G** and **36M** trigger the activation of autophagy due to increased cellular oxidative stress, which leads to oral cancer cell death.

### 2.5. Treatment with ***36M*** Decreased Malignant Ability and Angiogenesis of Cancer Cells

**Figure 5:**
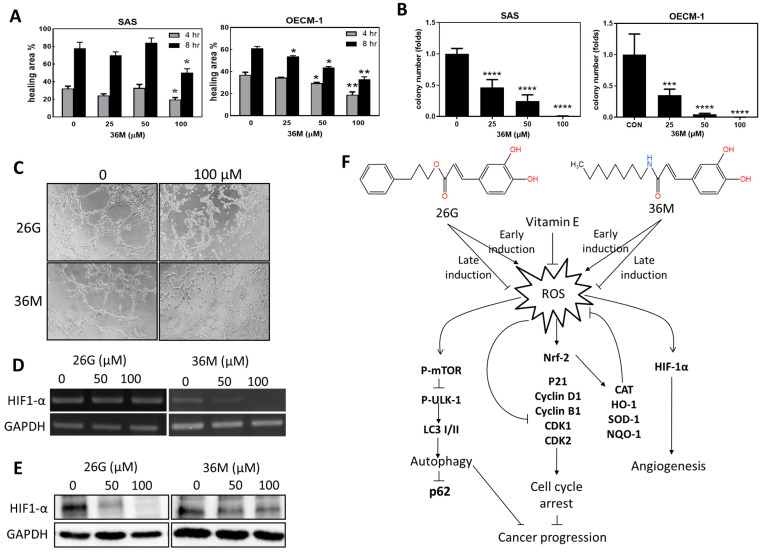
The **36M** inhibited cell migration and colony formation in the OSCC cell line and angiogenesis in the SVEC 4-10 cell line. (**A**) The SAS and OECM-1 cells were treated with 25, 50, and 100 μM of **36M** at different times. Cell migration was assessed by wound-healing assay. (**B**) The SAS or OECM-1 cells were treated with 25, 50, and 100 μM of **26G** and **36M** for 7 d, and colony formation was detected. (**C**) The SVEC 4-10 cells were treated with 50 and 100 μM of **36M** and **26G** for 24 h. The angiogenesis was observed and recorded at 200X magnification using bright-field microscopy. (**D**) *HIF1-α mRNA* expression was evaluated by RT-PCR. (**E**) HIF1-α protein expression was analyzed by western blot. (**F**) The mechanism of **26G** and **36M** against OSCC cells. * *p* < 0.05, ** *p* < 0.01, *** *p* < 0.001, **** *p* < 0.0001 compared to the vehicle control group. RT-PCR, reverse-transcriptase polymerase chain reaction; ROS, reactive oxygen species.

**26G** and **36M** induce cytotoxicity, cell cycle S or G2/M phase arrest, and autophagy in OSCC cells. Both SAS and OECM-1 cells were more sensitive to **36M** than to **26G**; therefore, the anti-OSCC potential of **36M** requires further investigation. The SAS cells treated with 100 μM **36M**, and the OECM-1 cells treated with 50 μM to 100 μM significantly reduced migration ability as compared with the corresponding control group after 4 or 8 h (Figure 5A). Furthermore, the colony formation in the SAS and OECM-1 cells was significant inhibited following the treatment with doses of 25 μM to 100 μM of **36M** (Figure 5B). The HIF1-α expression has been well demonstrated it played a vital role in cancer progression, angiogenesis, and metastasis [19,20]. The anti-angiogenic abilities of **26G** and 36 M were compared in SVEC 4-10 cells in vitro. After 100 μM **26G** and **36M** treatment, the microtube formation ability of SVEC 4-10 was inhibited. Partial microtube formation was still observed in the **26G** treatment group, but was completely absent in the **36M** treatment group (Figure 5C). *HIF1-α* mRNA and protein expression in SVEC4-10 cells were also analyzed. The SVEC4-10 cells treated with 50 μM and 100 μM **26G** or **36M** for 24 h, the **26G** suppressed HIF1-α protein only, and **36M** suppressed both *HIF1-α mRNA* and protein expression levels (Figure 5D,E).

The overall results and mechanisms of **26G** and **36M** suppression in OSCC cells are shown in Figure 5F. **26G** and **36M** early induction of ROS production, but late induction decreased ROS production in OSCC cells. This induced ROS promotes the expression of NRF-2 and activates intracellular antioxidant proteins (CAT, HO-1, SOD-1, and NQO-1) to reduce ROS in a positive regulatory manner. Simultaneously, ROS can cause cytotoxicity and cell-cycle arrest. Cell cycle-related proteins, including p21, cyclin D1, cyclin B1, and CDK2, were inhibited. In addition, ROS induced autophagy signaling, such as p-mTOR and p62 downregulation and p-ULK-1 and LC3 I/II upregulation. **26G** and **36M** induced changes in p62 and LC3 I/II, which were reversed by vitamin E pre-treatment. Finally, ROS activates HIF-1α expression. Treatment with **26G** and **36M** inhibited HIF-1α expression and interfered with tumor angiogenesis. Based on these results, we concluded that **26G** and **36M** are bioactive substances with anti-OSCC potential.

## 3. Discussion

In the present study, we demonstrated that **26G** and **36M** could decrease malignancy owing to their ability to induce oral cancer cell death through mTOR-ULK1-P62-LC3 signaling. CAPE has been used in the treatment of patients with oral cancer and has increased their survival rate in 5 years [25]. The IC50 of CAPE were 129.7 ± 4.2 μM and 159.2 ± 7.2 μM in SAS and OECM-1, respectively [26]. Compare to our result, the IC50 of CAPE (129.7 ± 4.2 μM) was closed to **26G** (54.0 μM) and **36M** (161.3 μM) in SAS cells. However, the cytotoxicity will be affected by cell density, treatment time, serum percentage in the medium, and analysis methods, etc. Herein, the lower level of caffeamide derivative **36M** treatment for 72 h presented a more potent ability to induce cell toxicity in cancer cells than the corresponding dose level of the CAPE derivative **26G** (Figure 1A,B). This study indicates that **36M** plays critical roles in preventing type 2 diabetes and oral microbes because of its broad resistance to acid, alkali, and high-temperature conditions [27,28]. Furthermore, **36M** showed more potent cell cytotoxicity than other CAPE derivatives [28]. The chemical structure revealed that **26G** was more hydrophilic than **36M** because the polarity of the aromatic group in **26G** was stronger than that of the **36M** alkyl group (Table 1). The solubility and diffusion rates of **26G** were low at **36M**, which could explain why the anticancer activity of **36M** was more potent than that of **26G** [28].

Based on the histograms shown on the right side of Figure 4C,D, it is evident that treatment with 25–100 μM of **26G** and **36M** led to a decrease in the expression of CDK1, CDK2, and cyclin B1 in OECM-1 and SAS cells. However, the treatment with 25 μM of **26G** induced cyclin B1 expression in SAS cells but not in OECM-1 cells. The expression of cyclin A2 showed inconsistency after treatment in both cell lines. This variability may be attributed to the different p53 statuses in OECM-1 and SAS cells. The tumor suppressor proteins p53 and p21 play a crucial role in inhibiting cell cycle progression. Mutations in the p53 gene have been detected in over 70% of human cancers. In particular, OECM-1 cells exhibited a point mutation at codon 173 (GTG to TTG), while SAS cells had a point mutation at codon 305 (GAG to a TAG stop codon) in their p53 genes. The molecular weights of the p53 protein were 53 kD in OECM-1 and 40 kD in SAS, indicating abnormal p53 functions in both cell lines. However, it is important to note that mutated p53 is involved in various cellular processes such as DNA repair, cell cycle regulation, programmed cell death, and senescence. Further investigation is required to understand the function of mutated p53 in OECM-1 and SAS cells [29].

LC3 is a well-known autophagy marker. When LC3-I is hydrolyzed by autophagy-related 4 (ATG4) from LC3-I, it is converted into LC3-II, termed the autophagosome, which fuses with the lysosome to form an autolysosome, leading to the degradation of cytoplasmic materials [30,31]. A previous study demonstrated that CAPE enhanced the autophagic response to the induction of cancer cell death by AMP-activated protein kinase (AMPK) inactivation in C6 glioma cells [32]. Another study showed that CAPE increased intracellular ROS levels by altering cellular oxidative and endoplasmic reticulum (ER) stress responses, triggering autophagy in SH-SY5Y cells [22]. In the present study, we also found that treatment with **26G** and **36M** led to the activation of autophagic signaling in SAS and OECM-1 cells (Figure 4). Moreover, phosphorylated p62 has been reported to play an important role in the autophagy response owing to its association with autophagy-lysosome degradation. During autophagy, p62 cannot be phosphorylated by optineurin (OPTN) or TANK-binding kinase 1 (TBK1), which prevents cytoplasmic materials from being degraded and deposited within the cytosol [33]. Herein, non-phosphorylated p62 protein was reduced by treatment with **26G** or **36M** for 24 h in oral cancer cells, remarkably increasing LC3-II expression (Figure 4), which suggests that the autolysosome was formed for cytoplasmic material degradation by **26G** and **36M** stimulation in a relatively late stage of autophagy.

In colon carcinoma, the inhibition of catalase to enhance intracellular levels of free radicals leads to cell death, which might provide a new concept to the clinical therapy of drugs [34]. In this study, cellular ROS levels were altered by **26G** and **36M** treatments in OSCC cells (Figure 3A,B). However, the oxidative-stress relative protein Nrf-2 and catalase were significantly decreased by **26G** and **36M** treatments (Figure 3C,D). A previous study showed that Nrf-2 is a nuclear transcription factor that increases the transcription and translation of the catalase protein in the cell [35]. In the antioxidant system, SOD is the primary defense enzyme for the chelation of superoxide (O2^•–^) to H_2_O_2_, and then, H_2_O_2_ is converted to H_2_O and O_2_ by catalase [36]. This defense system inhibits normal cells or leads to cancer cell death [36,37]. Hence, treatment with **26G** and **36M** caused unbalanced cellular oxidative stress, inducing cell death, which was not chelated by catalase upon correlation with cellular ROS (Figure 1 and Figure 3). Additionally, it is well-known that free radical generation causes lipid peroxidation and DNA damage or mitochondrial dysfunction to induce cell death in a dysfunctional antioxidant defense system [38]. Therefore, our results suggest that oral cancer cell death is linked to cellular free radical production and the downregulation of catalase protein expression under **26G** and **36M** treatments.

The accumulation of ROS in oral cancer cells was measured 24 h after **26G** and **36M** treatments. We found that ROS levels decreased due to cell death at 48 h (Figure 1 and Figure 3A,B). At this time, **26G** and **36M** treatments inhibited the expression of the transcriptional activator NRF2 from suppressing downstream anti-ROS gene transcription. CAT, HO-1, SOD-1, and NQO-1 protein expression decreased in a dose-dependent manner (Figure 3C,D). Therefore, **26G** and **36M** did not exert antioxidant activity in oral cancer cells but induced ROS generation in a short time. Increased cellular ROS levels activated apoptosis and autophagy in the tongue squamous cell carcinoma cells [39]. In the present study, the mTOR-ULK1-LC3 signaling pathway was upregulated by the **26G** and **36M** treatments at 100 μM for 24 h (Figure 4A,B). Vitamin E is an antioxidant that reduces the production of ROS. We used vitamin E to block intracellular ROS generation and decrease LC3 expression in SAS and OECM-1 cells under **26G** and **36M** treatments (Figure 4C–E). Our results indicated that **36M** increases autophagy by mediating mTOR-ULK1-P62-LC3 signaling in OSCC cells.

Approximately 90% of cancer-related deaths occur in patients with cancer cell metastasis [40]. Metastasis accelerates the mortality rate of patients with oral cancer [41]. In other words, preventing metastatic colonization could improve the survival rate of oral cancer patients, which may benefit clinical treatment [42].In fact, **26G** and **36M** inhibited malignant phenotypes, including cell migration, colony formation, and angiogenesis (Figure 5). Longitudinal clinical investigations showed that combined supplementation of herbal medicines with vitamin E efficiency reduced the mortality rate in lung cancer patients in 5 years [43,44]. Therefore, supplements containing bioactive herbal compounds may provide more efficient therapies for patients with cancer. Compounds **26G** and **36M** show potential as effective treatments for oral cancer by increasing oral cancer cell toxicity and preventing metastasis, which may increase the survival rate of patients with oral cancer after treatment.

## 4. Materials and Methods

### 4.1. Compound Purification

CAPE and caffeamide derivatives (**26G**, **36C**, **36H**, **36 K**, and **36M**) were synthesized and provided by Dr. Yueh-Hsiung Kuo, and the extraction processes were as reported previously [27,45]. Briefly, benzotriazol-1-yloxytris(dimethylamino)phosphonium hexafluorophosphate was dissolved in dichloromethane and added to a mixture of caffeic acid, phenethyl alcohol, corresponding amines, and triethylamine in dimethylformamide. The reaction mixture was evaporated under a vacuum and partitioned between ethyl acetate (EtOAc) and H_2_O. The EtOAc layer was washed with 3N HCl and 10% NaHCO_3_ and concentrated under a vacuum. All products were >95% purified via silica gel column chromatography (70–230 and 230–400 mesh, Merck, Burlington, MA, USA). All caffeamide derivatives were dissolved in dimethyl sulfoxide (DMSO) and stored at −20 °C.

### 4.2. Cell Culture

The two OSCC cell lines, SAS and OECM-1, were kindly provided by Professor Hsi-Feng Tu from the National Yang-Ming Chiao Tung University. SAS cells were cultured in Dulbecco’s modified Eagle’s medium (DMEM) containing 10% fetal bovine serum (FBS) and 1% penicillin and streptomycin. OECM-1 cells were cultured in a RPMI-1640 medium containing 10% FBS and 1% penicillin and streptomycin solution. The mouse vascular endothelial cell line SVEC4-10 was purchased from the Bioresource Collection and Research Center (BCRC: 60220, Hsinchu, Taiwan). The SVEC4-10 cells were cultured in DMEM containing 10% FBS, 4 mM glutamine, and 1% penicillin and a streptomycin mixture solution. All the cell lines were cultured at 37 °C in a 5% CO_2_ atmosphere, as described previously [46,47]. When the cells reached approximately 90% confluence, they were treated with **26G** or caffeamide derivatives at different times and doses depending on the experimental design. DMSO was used as the vehicle control.

### 4.3. Cell Viability

SAS and OECM-1 cells (5 × 10^3^ cells) were inoculated into each well of 96-well tissue culture plates and cultured at 37 °C under 5% CO_2_ for 20–24 h. Cells were treated with the vehicle or various concentrations of CAPE and caffeamide derivatives in 10% FBS culture media for 24, 48, and 72 h. At the end of the experiment, the 3-(4,5-dimethylthiazol-2-yl)-2,5-diphenyltetrazolium bromide (MTT) assay was performed according to the previously reported protocol [48,49].

### 4.4. Cell cycle Arrest Determination

SAS and OECM-1 cells (5 × 10^5^ cells) were cultured in 6 cm cell culture dishes for 20–24 h. The cells were then treated with the vehicle or various concentrations of **26G** or **36M** for 48 h. Next, the cells were fixed with 70% ethanol at −20 °C overnight and stained with propidium iodide/Triton X-100 mixtures at room temperature (~25 °C) for 30 min. The cell cycle distribution was determined using flow cytometry (BD Biosciences, Franklin Lakes, NJ, USA) [29].

### 4.5. Intracellular ROS Level

SAS and OECM-1 cells (5 × 10^5^ cells) were cultured in 6 cm cell culture dishes for 20–24 h. The cells were then treated with vehicle or various concentrations of **26G** or **36M** for 48 h. Subsequently, the cells were incubated with 20 µM 5-(and 6)-Carboxy-2′,7′-dichlorodihydrofluorescein diacetate (carboxy-H2DCFDA) in a serum-free medium at 37 °C for 45 min in the dark. Finally, cellular ROS levels were detected using a flow cytometer (Thermo Fisher Scientific, Waltham, MA, USA), and fluorescence images were captured using a fluorescence microscope (Eclipse TS100, Nikon, Minato, Tokyo, Japan).

### 4.6. Western Blot

Western blotting was performed using 50 μg proteins, as described previously [50]. Cell lysates were electrophoresed on a 10% polyacrylamide gel and transferred to a polyvinylidene difluoride (PVDF) membrane. The membranes were blocked with 5% nonfat milk for 1 h and then incubated with primary antibodies at 4 °C overnight (Supplementary Table S1). After washing, the membranes were incubated with a secondary antibody (Abcam, Cambridge, UK) for 1 h. GAPDH was used as the internal control. Chemiluminescence imaging was performed using the SuperSignal West Femto chemiluminescent substrate (Thermo Scientific, Rockford, IL, USA). Densitometric estimates were quantified using ImageJ software (National Institutes of Health, Bethesda, MD, USA).

### 4.7. Wound Healing

SAS and OECM-1 cells (5 × 10^5^ cells) were incubated in 6 cm cell culture dishes for 20–24 h. Sterile 200 µL pipette tips were used to scratch a line in the middle of each well. The cells were treated with vehicle or various concentrations of **26G** or **36M**, and observed at different time points. The wounded area was observed and photographed using a microscope and quantified by wound width using ImageJ software (National Institutes of Health, Bethesda, MD, USA) [29].

### 4.8. Colony Formation

For the colony formation assay, SAS 2 × 10^3^ cells/3 mL and OECM-1 5 × 10^2^ cells/3 mL cells were incubated in each well of 6-well tissue culture plates and cultured at 37 °C under 5% CO_2_ for 20–24 h. Cells were treated with the vehicle or various concentrations of **26G** or **36M** in a medium supplemented with 10% FBS for 24 h. The old medium was replaced with a fresh culture medium, and cells were then cultured for another 7 days to allow single cells to form colonies. At the end of the experiment, the colonies were fixed in methanol and stained with 0.01% crystal violet for 20 min at room temperature. Subsequently, the colonies were randomly photographed in 5 fields per well. Colonies containing > 50 cells were counted in each field. The number of colonies formed in each well was calculated [29].

### 4.9. Tube Formation Assay

Matrigel (50 μL; BD Biosciences; 354234) was added into 96-well plates and incubated at 37 °C for 1 h for gel formation. Next, 1 × 10^5^ SVEC4-10 cells were incubated on colloidal Matrigel and treated with the vehicle or various concentrations of **26G** and **36M**. Random photographs of tube formation were taken for each well for 2–4 h after **26G** and **36M** stimulation [47].

### 4.10. RT-PCR Analysis

Reverse transcription–polymerase chain reaction (RT-PCR) was used to analyze the mRNA expression. RNA extraction and RT-PCR were conducted according to previously used protocols [46]. The oligonucleotide primers used corresponded to *HIF-1α* (forward, 5′-GACACAGATTTAGACTTGGAG-3′; reverse, 5′-TGGGTAGGAGATGGAGATGC-3′) and *glyceraldehyde-3-phosphate dehydrogenase* (*GAPDH*; forward, 5′-ACACCCACTCCTCCACCTTT-3′; reverse. 5′-TAGCCAAATTCGTTGTCATACC-3′). The *HIF-α mRNA* expression signals were normalized with those of *GAPDH*.

### 4.11. Statistical Analysis

All experiments were performed at least three times independently. All experimental results were analyzed using Student’s *t*-test using SPSS software (IBM, Armonk, NY, USA), where a *p*-value of < 0.05 represents a significant difference.

## 5. Conclusions

We concluded that ROS played a dominant role in inducing cell death under **26G** and **36M** treatments by regulating the mTOR-ULK1-P62-LC3 signaling pathway. Thus, **26G** and **36M** might be used to treat patients with oral cancer.

## Figures and Tables

**Table 1:** IC50 of CAPE derivatives and caffeamide derivatives in SAS cell line.

Compounds	Chemical Structure	Molecular Weight	IC50 (μM)
**26G**	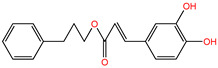	298.33	54.0
**36C**	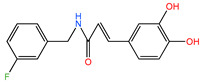	287.29	335.1
**36H**	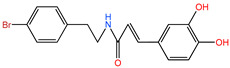	362.22	393.9
**36K**	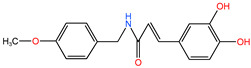	299.32	347.9
**36M**	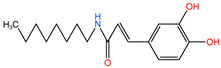	291.32	161.3

## Data Availability

Data are contained within the article.

## Supplementary Materials

The following supporting information can be downloaded at https://www.mdpi.com/article/10.3390/ijms24129819/s1.

## References

[1] Mehrtash H., Duncan K., Parascandola M., David A., Gritz E.R., Gupta P.C., Mehrotra R., Amer Nordin A.S., Pearlman P.C., Warnakulasuriya S. (2017). Defining a global research and policy agenda for betel quid and areca nut. Lancet. Oncol..

[2] Mehanna H., Beech T., Nicholson T., El-Hariry I., McConkey C., Paleri V., Roberts S. (2013). Prevalence of human papillomavirus in oropharyngeal and nonoropharyngeal head and neck cancer--systematic review and meta-analysis of trends by time and region. Head Neck.

[3] Ahmad P., Nawaz R., Qurban M., Shaikh G.M., Mohamed R.N., Nagarajappa A.K., Asif J.A., Alam M.K. (2021). Risk factors associated with the mortality rate of oral squamous cell carcinoma patients: A 10-year retrospective study. Medicine.

[4] Ong T.K., Murphy C., Smith A.B., Kanatas A.N., Mitchell D.A. (2017). Survival after surgery for oral cancer: A 30-year experience. Br. J. Oral. Maxillofac. Surg..

[5] Hossain R., Quispe C., Khan R.A., Saikat A.S.M., Ray P., Ongalbek D., Yeskaliyeva B., Jain D., Smeriglio A., Trombetta D. (2022). Propolis: An update on its chemistry and pharmacological applications. Chin. Med..

[6] Burdock G.A. (1998). Review of the biological properties and toxicity of bee propolis (propolis). Food Chem. Toxicol..

[7] Khurshid Z., Naseem M., Zafar M.S., Najeeb S., Zohaib S. (2017). Propolis: A natural biomaterial for dental and oral healthcare. J. Dent. Res. Dent. Clin. Dent. Prospect..

[8] Firat F., Ozgul M., Turkoz Uluer E., Inan S. (2019). Effects of caffeic acid phenethyl ester (CAPE) on angiogenesis, apoptosis and oxidative stress in various cancer cell lines. Biotech. Histochem..

[9] Yu H.J., Shin J.A., Yang I.H., Won D.H., Ahn C.H., Kwon H.J., Lee J.S., Cho N.P., Kim E.C., Yoon H.J. (2017). Apoptosis induced by caffeic acid phenethyl ester in human oral cancer cell lines: Involvement of Puma and Bax activation. Arch. Oral. Biol..

[10] Chung L.C., Chiang K.C., Feng T.H., Chang K.S., Chuang S.T., Chen Y.J., Tsui K.H., Lee J.C., Juang H.H. (2017). Caffeic acid phenethyl ester upregulates N-myc downstream regulated gene 1 via ERK pathway to inhibit human oral cancer cell growth in vitro and in vivo. Mol. Nutr. Food Res..

[11] Peng C.Y., Yang H.W., Chu Y.H., Chang Y.C., Hsieh M.J., Chou M.Y., Yeh K.T., Lin Y.M., Yang S.F., Lin C.W. (2012). Caffeic Acid phenethyl ester inhibits oral cancer cell metastasis by regulating matrix metalloproteinase-2 and the mitogen-activated protein kinase pathway. Evid.-Based Complement. Altern. Med..

[12] Celli N., Dragani L.K., Murzilli S., Pagliani T., Poggi A. (2007). In vitro and in vivo stability of caffeic acid phenethyl ester, a bioactive compound of propolis. J. Agric. Food Chem..

[13] Celli N., Mariani B., Dragani L.K., Murzilli S., Rossi C., Rotilio D. (2004). Development and validation of a liquid chromatographic-tandem mass spectrometric method for the determination of caffeic acid phenethyl ester in rat plasma and urine. J. Chromatogr..

[14] Natarajan K., Singh S., Burke T.R., Grunberger D., Aggarwal B.B. (1996). Caffeic acid phenethyl ester is a potent and specific inhibitor of activation of nuclear transcription factor NF-kappa B. Proc. Natl. Acad. Sci. USA.

[15] Kuo Y.Y., Huo C., Lin C.Y., Lin H.P., Liu J.S., Wang W.C., Chang C.R., Chuu C.P. (2019). Caffeic acid phenethyl ester suppresses androgen receptor signaling and stability via inhibition of phosphorylation on Ser81 and Ser213. Cell. Commun. Signal. CCS.

[16] Pagnan A.L., Pessoa A.S., Tokuhara C.K., Fakhoury V.S., Oliveira G.S.N., Sanches M.L.R., Inacio K.K., Ximenes V.F., Oliveira R.C. (2022). Anti-tumour potential and selectivity of caffeic acid phenethyl ester in osteosarcoma cells. Tissue Cell..

[17] Balc-Okcanoğlu T., Yilma-Susluer S., Kayabasi C., Ozme-Yelken B., Biray-Avci C., Gunduz C. (2021). The effect of caffeic acid phenethyl ester on cell cycle control gene expressions in breast cancer cells. Mol. Biol. Res. Commun..

[18] Day R.M., Suzuki Y.J. (2006). Cell proliferation, reactive oxygen and cellular glutathione. Dose Response.

[19] Lu J.M., Lin P.H., Yao Q., Chen C. (2010). Chemical and molecular mechanisms of antioxidants: Experimental approaches and model systems. J. Cell. Mol. Med..

[20] Zaidieh T., Smith J.R., Ball K.E., An Q. (2019). ROS as a novel indicator to predict anticancer drug efficacy. BMC Cancer.

[21] Villalpando-Rodriguez G.E., Gibson S.B. (2021). Reactive Oxygen Species (ROS) Regulates Different Types of Cell Death by Acting as a Rheostat. Oxid. Med. Cell. Longev..

[22] Tomiyama R., Takakura K., Takatou S., Le T.M., Nishiuchi T., Nakamura Y., Konishi T., Matsugo S., Hori O. (2018). 3,4-dihydroxybenzalacetone and caffeic acid phenethyl ester induce preconditioning ER stress and autophagy in SH-SY5Y cells. J. Cell. Physiol..

[23] Chang H., Wang Y., Yin X., Liu X., Xuan H. (2017). Ethanol extract of propolis and its constituent caffeic acid phenethyl ester inhibit breast cancer cells proliferation in inflammatory microenvironment by inhibiting TLR4 signal pathway and inducing apoptosis and autophagy. BMC Complement. Altern. Med..

[24] Vijayakurup V., Spatafora C., Tringali C., Jayakrishnan P.C., Srinivas P., Gopala S. (2014). Phenethyl caffeate benzoxanthene lignan is a derivative of caffeic acid phenethyl ester that induces bystander autophagy in WiDr cells. Mol. Biol. Rep..

[25] Kuo Y.Y., Jim W.T., Su L.C., Chung C.J., Lin C.Y., Huo C., Tseng J.C., Huang S.H., Lai C.J., Chen B.C. (2015). Caffeic Acid phenethyl ester is a potential therapeutic agent for oral cancer. Int. J. Mol. Sci..

[26] Lee Y.T., Don M.J., Hung P.S., Shen Y.C., Lo Y.S., Chang K.W., Chen C.F., Ho L.K. (2005). Cytotoxicity of phenolic acid phenethyl esters on oral cancer cells. Cancer Lett..

[27] Wu M.Y., Liu C.C., Lee S.C., Kuo Y.H., Hsieh T.J. (2022). N-Octyl Caffeamide, a Caffeic Acid Amide Derivative, Prevents Progression of Diabetes and Hepatic Steatosis in High-Fat Diet Induced Obese Mice. Int. J. Mol. Sci..

[28] Shih Y.H., Hsia S.M., Chiu K.C., Wang T.H., Chien C.Y., Li P.J., Kuo Y.H., Shieh T.M. (2022). In Vitro Antimicrobial Potential of CAPE and Caffeamide Derivatives against Oral Microbes. Int. J. Mol. Sci..

[29] Hsia S.M., Yu C.C., Shih Y.H., Yuanchien Chen M., Wang T.H., Huang Y.T., Shieh T.M. (2016). Isoliquiritigenin as a cause of DNA damage and inhibitor of ataxia-telangiectasia mutated expression leading to G2/M phase arrest and apoptosis in oral squamous cell carcinoma. Head. Neck.

[30] Yim W.W., Mizushima N. (2020). Lysosome biology in autophagy. Cell. Discov..

[31] Mizushima N., Komatsu M. (2011). Autophagy: Renovation of cells and tissues. Cell.

[32] Yu S.H., Kao Y.T., Wu J.Y., Huang S.H., Huang S.T., Lee C.M., Cheng K.T., Lin C.M. (2011). Inhibition of AMPK-associated autophagy enhances caffeic acid phenethyl ester-induced cell death in C6 glioma cells. Planta Med..

[33] Matsumoto G., Shimogori T., Hattori N., Nukina N. (2015). TBK1 controls autophagosomal engulfment of polyubiquitinated mitochondria through p62/SQSTM1 phosphorylation. Hum. Mol. Genet..

[34] Kankala R.K., Kuthati Y., Liu C.-L., Mou C.-Y., Lee C.-H. (2015). Killing cancer cells by delivering a nanoreactor for inhibition of catalase and catalytically enhancing intracellular levels of ROS. RSC Adv..

[35] Chen J., Zhang Z., Cai L. (2014). Diabetic cardiomyopathy and its prevention by nrf2: Current status. Diabetes Metab. J..

[36] Hamada N., Fujimichi Y., Iwasaki T., Fujii N., Furuhashi M., Kubo E., Minamino T., Nomura T., Sato H. (2014). Emerging issues in radiogenic cataracts and cardiovascular disease. J. Radiat. Res..

[37] Su E.Y., Chu Y.L., Chueh F.S., Ma Y.S., Peng S.F., Huang W.W., Liao C.L., Huang A.C., Chung J.G. (2019). Bufalin Induces Apoptotic Cell Death in Human Nasopharyngeal Carcinoma Cells through Mitochondrial ROS and TRAIL Pathways. Am. J. Chin. Med..

[38] Ryter S.W., Kim H.P., Hoetzel A., Park J.W., Nakahira K., Wang X., Choi A.M. (2007). Mechanisms of cell death in oxidative stress. Antioxid. Redox Signal..

[39] Xue D.F., Pan S.T., Huang G., Qiu J.X. (2020). ROS enhances the cytotoxicity of cisplatin by inducing apoptosis and autophagy in tongue squamous cell carcinoma cells. Int. J. Biochem. Cell. Biol..

[40] Seyfried T.N., Huysentruyt L.C. (2013). On the origin of cancer metastasis. Crit. Rev. Oncog..

[41] Ho A.S., Kim S., Tighiouart M., Gudino C., Mita A., Scher K.S., Laury A., Prasad R., Shiao S.L., Van Eyk J.E. (2017). Metastatic Lymph Node Burden and Survival in Oral Cavity Cancer. J. Clin. Oncol. Off. J. Am. Soc. Clin. Oncol..

[42] Massagué J., Obenauf A.C. (2016). Metastatic colonization by circulating tumour cells. Nature.

[43] McCulloch M., Broffman M., van der Laan M., Hubbard A., Kushi L., Kramer A., Gao J., Colford J.M. (2011). Lung cancer survival with herbal medicine and vitamins in a whole-systems approach: Ten-year follow-up data analyzed with marginal structural models and propensity score methods. Integr. Cancer Ther..

[44] Yeh M.H., Wu H.C., Lin N.W., Hsieh J.J., Yeh J.W., Chiu H.P., Wu M.C., Tsai T.Y., Yeh C.C., Li T.M. (2020). Long-term use of combined conventional medicine and Chinese herbal medicine decreases the mortality risk of patients with lung cancer. Complement. Ther. Med..

[45] Tsai T.H., Yu C.H., Chang Y.P., Lin Y.T., Huang C.J., Kuo Y.H., Tsai P.J. (2017). Protective Effect of Caffeic Acid Derivatives on tert-Butyl Hydroperoxide-Induced Oxidative Hepato-Toxicity and Mitochondrial Dysfunction in HepG2 Cells. Molecules.

[46] Lin N.C., Shih Y.H., Chiu K.C., Li P.J., Yang H.W., Lan W.C., Hsia S.M., Wang T.H., Shieh T.M. (2022). Association of rs9679162 Genetic Polymorphism and Aberrant Expression of Polypeptide N-Acetylgalactosaminyltransferase 14 (GALNT14) in Head and Neck Cancer. Cancers.

[47] Shih Y.H., Chang K.W., Chen M.Y., Yu C.C., Lin D.J., Hsia S.M., Huang H.L., Shieh T.M. (2013). Lysyl oxidase and enhancement of cell proliferation and angiogenesis in oral squamous cell carcinoma. Head. Neck.

[48] Shih Y.H., Lin D.J., Chang K.W., Hsia S.M., Ko S.Y., Lee S.Y., Hsue S.S., Wang T.H., Chen Y.L., Shieh T.M. (2014). Evaluation physical characteristics and comparison antimicrobial and anti-inflammation potentials of dental root canal sealers containing hinokitiol in vitro. PLoS ONE.

[49] Asadi-Samani M., Rafieian-Kopaei M., Lorigooini Z., Shirzad H. (2018). A screening of growth inhibitory activity of Iranian medicinal plants on prostate cancer cell lines. Biomedince.

[50] Shih Y.H., Chiu K.C., Wang T.H., Lan W.C., Tsai B.H., Wu L.J., Hsia S.M., Shieh T.M. (2021). Effects of melatonin to arecoline-induced reactive oxygen species production and DNA damage in oral squamous cell carcinoma. J. Med. Assoc..

