# Supplementary material for: Caffeic Acid Phenethyl Ester and Caffeamide Derivatives Suppress Oral Squamous Cell Carcinoma Cells
Source: Int J Mol Sci. 2023 Jun 6;24(12):9819. doi: 10.3390/ijms24129819 (PMC10298160; doi:10.3390/ijms24129819)
Supplement: Supplementary file 1 [file ijms-24-09819-s001.zip › ijms-2374739-supplementary.pdf]

Supplementary Table S1. List of antibodies for Western blot.

**Antibody**	**Brand**	**Cat. Number,**	**Dilution**
cyclin D1	Cell Signaling	2978	1:1000
cyclin A2	Cell Signaling	4656	1:1000
cyclin B1	Cell Signaling	4138	1:1000
CDK2	Cell Signaling	2546	1:1000
CDK1	Cell Signaling	9112	1:1000
P21	Cell Signaling	2947	1:1000
Nrf-2	Cell Signaling	12721	1:1000
Catalase	Santa Cruz	sc-271803	1:1000
HO-1	Santa Cruz	sc-136960	1:1000
SOD-1	Santa Cruz	sc-515404	1:1000
NQO1	Santa Cruz	sc-32793	1:1000
p-mTOR	Cell Signaling	2971	1:1000
p-ULK1	Cell Signaling	6888	1:1000
P62,	Proteintech	18420-1-AP	1:1000
LC-3	Cell Signaling	4108	1:1000
HIF1-α	Santa Cruz	sc-53546	1:1000
GAPDH	Novus Biologicals	NB300-221	1:10000
